# A randomised clinical trial comparing 35 Hz versus 50 Hz frequency stimulation effects on hand motor recovery in older adults after stroke

**DOI:** 10.1038/s41598-021-88607-8

**Published:** 2021-04-28

**Authors:** Trinidad Sentandreu-Mañó, José M. Tomás, J. Ricardo Salom Terrádez

**Affiliations:** 1 Department of Physical Therapy, University of Valencia, 46610 Valencia, Spain; 2 FISABIO, 46020 Valencia, Spain; 3 Department of Methodology for the Behavioral Sciences, University of Valencia, 46010 Valencia, Spain; 4 Physical Medicine and Rehabilitation Service, Doctor Peset University Hospital, 46017 Valencia, Spain

**Keywords:** Health care, Medical research, Neurology

## Abstract

More solid data are needed regarding the application of neuromuscular electrical stimulation (NMES) in the paretic hand following a stroke. A randomised clinical trial was conducted to compare the effects of two NMES protocols with different stimulation frequencies on upper limb motor impairment and function in older adults with spastic hemiparesis after stroke. Sixty nine outpatients were randomly assigned to the control group or the experimental groups (NMES with 50 Hz or 35 Hz). Outcome measures included motor impairment tests and functional assessment. They were collected at baseline, after 4 and 8 weeks of treatment, and after a follow-up period. NMES groups showed significant changes (p < 0.05) with different effect sizes in range of motion, grip and pinch strength, the Modified Ashworth Scale, and the muscle electrical activity in the extensors of the wrist. The 35 Hz NMES intervention showed a significant effect on Barthel Index. Additionally, there were no significant differences between the groups in the Box and Block Test. Both NMES protocols proved evidence of improvements in measurements related to hand motor recovery in older adults following a stroke, nevertheless, these findings showed that the specific stimulation frequency had different effects depending on the clinical measures under study.

## Introduction

Non-fatal stroke constitutes one of the major causes of disability in old age^1,2^. Forecasts indicate that the total cost resulting from this illness will triple between 2012 and 2030, with a major part of the projected increase in costs deriving from older adults^3^. Furthermore, recent data from the American Heart Association indicates that the oldest population have greatest disabilities, receive less evidenced-based care, and are less likely to be discharged to their residences^3^.

A large number of stroke survivors, some 69 to 80%, initially present impairments in the upper limbs^4^. Upper extremity hemiparesis is considered to be one of the most frequent conditions underlying stroke-induced disability^5^, and between 55 and 75% of cases show significant residual deficits^6^. Among these deficits, those pertaining to the hand are those which persist the most. Thus, functions such as grasping, holding and manipulating objects are deficient in a high percentage of patients between 3 and 6 months after stroke, and full functional recovery of the hand has only been documented in between 5 and 20% of these subjects^7^. These impairments will impact functional motor ability and quality of life, and exert a great economic, social and personal toll.

The available research emphasises the need for interdisciplinary and multimodal approaches in the rehabilitation of the paretic hand^8^. Neuromuscular electrical stimulation (NMES) is one of the techniques proposed for upper limb recovery following a stroke^9–12^. Based on the use of electrical current to produce repetitive contractions of muscles it helps in restoring or assisting movements that would not otherwise occur because of hemiparesis. This treatment has certain characteristics labeled as important components of an effective intervention to promote motor recovery after stroke^8,13^, such as: repetitive movement, intensive practice, proprioceptive and exteroceptive input, visual feedback, and subject’s attention. In addition, it is also a low-cost and safe technique, easy to apply, without significantly increasing demands on stretched therapist’s time, and allows intensive home applications.

The specific mechanisms underlying this intervention are complex and unclear, but findings suggest that improvements could be mediated by local and central effects. At the local level, reference has been made to changes in muscular strength, modification of viscoelastic characteristics, and increase of blood flow^14,15^. Mechanisms also could involve increased presynaptic inhibition of muscle spindle reflex activity^16^. NMES could influence cortical plasticity^17,18^. It is hypothesised that either concurrent stimulation of afferent fibres, or antidromic discharge triggered by stimulation lead to enhanced synaptic remodelling, but evidence is still lacking. Concomitant physiologic changes in the brain including activation of primary sensory and motor areas, and the supplementary motor area, a reduction of intracortical inhibition, and an increased amplitude of motor-evoked potentials could be associated with NMES^19^.

Regarding previous systematic reviews and meta-analysis, Eraifej et al.^20^ found significant benefit from functional electrical estimulation applied within 2 months of stroke on activities of daily living. However, high quality large-scale randomised controlled trials are needed to obtain firm conclusions about its effectiveness or the optimum therapeutic window. They also stated the need of future research to identify the optimal parameters in order to standardise treatment for future studies. Nascimento et al.^21^ concluded that cyclical electrical stimulation increases strength and improves activity after stroke but there are insufficient data to provide evidence regarding the effect of different doses or modalities of electrical stimulation. Concerning the stimulation parameters required, there are still discussions in the literature, as some previous studies pointed out^11,17,18,22^. Therefore, more evidence is required to establish the most efficient NMES protocols (current, dose, parameters, modality, muscle targeted, etc) and to define the characteristics of the candidates for this type of treatment.

In this regard, the parameters of the electrical current used are currently subject of debate, and more data are needed to optimise the application of electrostimulation. Some reviews have pointed out that the comparison of stimulation parameters is complicated due to the disparity of variables, or the omission of data^23,24^. On one hand, it has been noted that one of the factors associated with a bad treatment result could relate to the use of inappropriate parameters^25^, the choice of which could result in different neurophysiological responses or, on the other hand, that the parameters might not be decisive in the effect of the intervention, but it has not yet been possible to confirm their clinical relevance^17^. A recent study published in 2020 was aimed to review the efficacy of the various parameters of application on the NMES in dysphagia generated after stroke. This concludes that the greatest efficacy of the technique was reached when applied at 60–80 Hz, 700 μs of pulse duration, at the motor intensity threshold, and in sessions of 20–30 min. The authors stated that there must be an adequate combination of between frequency and intensity to reach a quality muscular contraction, stimulating mainly type II fibres and adjusting to the needs of the target muscles in dysphagia^26^. Stimulation frequency is one of the basic parameters to be programmed in the NMES equipment. Regarding the recovery of the upper limb, the most commonly used parameters have been low frequency rectangular biphasic currents, with frequency values varying from 20 to 100 Hz. It is unknown which frequencies are the most effective within this wide range, although the most used in upper limb distal applications of clinical trials aimed at the motor recovery of adults after stroke range from 35 to 50 Hz^23^. Two studies have been found which examined the effectiveness of electrostimulation in relation to stimulation frequency, but they were limited to fine motor control and muscular fatigue in the hand^27,28^. Additionally, despite the enormous impact that stroke has on older adults, there is a complete paucity of literature directed expressly at old age with distinct physiological characteristics and the presence of comorbidity, which could entail the need for specific adaptations in their healthcare^29^.

Therefore, considering the bibliographic background, it was decided to conduct a trial with the objective of assessing and comparing the effect of two NMES protocols with different stimulation frequencies (35 Hz versus 50 Hz) on hand motor impairment (range of motion, grip and pinch strength, muscle tone and muscle electrical activity) and upper limb function (manual dexterity and functional independence) in older adults with spastic hemiparesis after stroke.

## Results

### Dropouts, baseline characteristics, and adverse events

**Figure 1:**
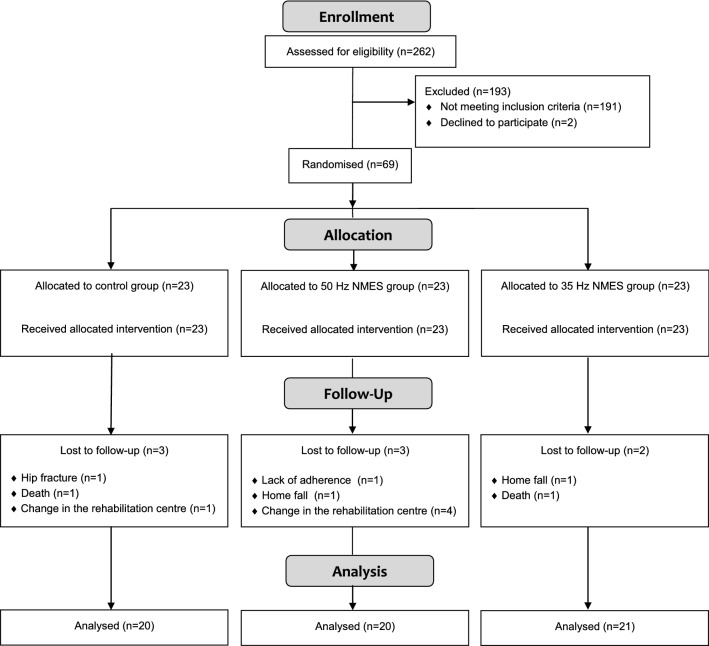
Participant flowchart according to CONSORT 2010.

Among the 262 eligible patients, the 69 participants selected were randomly placed into one of the three groups, resulting in the three balanced groups of 23 participants. There were eight dropouts during the study period: two patients died because of exacerbations of their chronic diseases, one patient had a hip fracture, two patients were excluded because of a change in the rehabilitation centre, two patients needed a rest because of a fall that happened at home, and finally, one patient was excluded because of a lack of adherence to treatment. Therefore, the final sample analysed was 61, 20 in the control group, 20 in the 50 Hz treatment group, and 21 in the 35 Hz treatment group (see Fig. 1).

With respect to the descriptive characteristics of the sample, 60.7% of the sample were men. Mean age was 70.95 years (SD = 7.18 years), with a range from 60 to 86 years, and an average body mass index of 25.32 (SD = 2.62). The average time elapsed since the stroke to the beginning of the study was 5.77 months (SD = 3.16), with a minimum of 1 month and a maximum of 15 months. Mean score in the Mini-Mental State Examination (MMSE) was 27.43 (SD = 3.56), 72.1% of subjects had suffered ischemic strokes, 49.2% had the right side of the body affected, 11.5% presented mild aphasia, 96.7% reported the right hand as the dominant hand, 54.1% had shoulder motor control, 44.3% had hypertension, 31.1% had diabetes, and finally, 45.9% were ex-smokers. Baseline characteristics of the control and experimental groups are presented in Table 1. There were no statistically significant differences in baseline variables among groups (p > 0.05) (Table 1). There were no significant differences in the number of treatment sessions completed between the groups (p > 0.05). There were no adverse events related to the trial. One subject felt forearm pain during stimulation session, but it was resolved by decreasing the intensity of the current.

**Table 1:** Means and standard deviations or percentages at baseline of the control and experimental groups and their tests for statistical differences.

Variable	50 Hz NMES group (n = 20)	35 Hz NMES group (n = 21)	Control group (n = 20)	P value
Gender (% male)	65	51.7	60	0.948
Age (years)	71.25 (6.99)	70.14 (7.25)	71.50 (7.56)	0.816
BMI (kg/m^2^)	25.33 (2.35)	24.49 (2.31)	26.19 (2.99)	0.114
Time post-stroke (months)	5.30 (2.13)	6.19 (3.92)	5.80 (3.24)	0.673
MMSE	27.10 (3.89)	27.81 (3.34)	27.35 (3.59)	0.816
Stroke (% ischemic)	75	61.9	80	0.45
Hemiparesis (% right)	55	52.4	40	0.648
Aphasia (%)	20	9.5	5	0.373
Dominant hand (% right)	100	95.2	95	1
Shoulder control motor (%)	65	47.6	50	0.523
Hypertension (%)	40	47.6	45	0.949
Diabetes (%)	35	23.8	35	0.735
Ex-smokers (%)	60	33.3	45	0.26
Pain (%)	70	71.4	55	0.501
Wrist resting angle (º)	− 10.50 (10.87)	− 10.48 (9.74)	− 5.50 (5.60)	0.138
Wrist active extension (º)	13.10 (6.54)	17.57 (11.89)	19.25 (18.01)	0.309
Wrist passive extension (º)	36.25 (14.77)	46.43 (12.56)	40.75 (16.65)	0.094
MCP resting angle (º)	− 35.00 (15.64)	− 37.14 (13.93)	− 29.75 (17.28)	0.308
MCP active extension (º)	6.00 (9.95)	15.24 (13.83)	12.75 (12.19)	0.05
Grip strength (kg_f_)	3.81 (3.01)	5.83 (4.70)	4.80 (6.30)	0.417
Pinch strength (kg_f_)	1.67 (1.55)	2.44 (2.12)	2.09 (2.39)	0.485
MAS score for wrist flexors	2.05 (0.99)	2.24 (0.77)	1.85 (0.75)	0.345
MAS score for MCP flexors	1.65 (1.23)	1.81 (0.98)	1.35 (1.04)	0.396
Peak EMG amplitude extensors (µV)	62.75 (52.77)	73.24 (42.40)	89.95 (112.86)	0.521
ACR extensors (%)	45.25 (12.52)	39.70 (12.65)	42.35 (12.05)	0.366
BBT	5.55 (5.52)	6.05 (7.71)	8.55 (10.05)	0.447
Barthel Index	59.50 (13.85)	60.71 (14.26)	58.25 (17.11)	0.873

*NMES* Neuromuscular electrical stimulation, *BMI* Body mass index, *MMSE* Mini-Mental State Examination, *MCP* Metacarpophalangeal, *MAS* Modified Ashworth Scale, *ACR* co-activation ratio of the antagonist, *BBT* Box and Block Test.

### Effects on range of motion

A Repeated Measures MANOVA (RM-MANOVA) was calculated for the five measures of range of motion for the three groups (control, 35 Hz NMES and 50 Hz NMES) in the four time points. The results of the multivariate effect of the interaction among these factors (range of motion, group and time) were statistically significant: Pillai’s trace = .754, F = 2.42, p = .001, η^2^ = .377. Once the RM-MANOVA gave significant results follow-up repeated measures ANOVAs were performed for each dependent variable. There was a statistically significant group per time interaction on the resting angle (F = 7.18; p < .001; η^2^ = .20), active extension (F = 17.31; p < .001; η^2^ = .37), and passive extension (F = 9.96; p< .001; η^2^ = .26) of the wrist, as well as in the resting angle (F = 5; p< .001; η^2^ = .15), and active extension (F = 3.25; p = .026; η^2^ = .10) of the metacarpophalangeal joints (MCP). Table 2 shows the means, standard deviations, mean differences of baseline measures against all other time points, as well as Cohen’s d for these differences for all these dependent variables, and their statistical significance. With all the detailed information in Table 2, a general pattern of mean differences arises. Both 35 Hz and 50 Hz NMES had consistent effects in improving the range of motion for all dependent variables. Control group only presented a significant gain in the case of wrist passive extension, but was ineffective for all other measures of range of motion. Another clear result is that gains produced by the 35 Hz were larger than those in the 50 Hz group.

**Table 2:** Means, standard deviations, mean differences, confidence intervals and effect sizes of the variables related to the range of motion in all the time points.

Measure		Control group (n = 20)			35 Hz NMES (n = 21)			50 Hz NMES (n = 20)		
Range of motion (º)	Testing time	Mean (SD)	MD (CI 95%)	ES (d)	Mean (SD)	MD (CI 95%)	ES (d)	Mean (SD)	MD (CI 95%)	ES (d)
Wrist resting angle	Baseline	− 5.50 (5.60)			− 10.48 (9.74)			− 10.50 (10.87)		
	1-month	− 4.75 (4.99)	− 0.75 (− 2.92, 1.42)	− 0.14	− 5.24 (9.01)	− 5.23 (− 7.35, − 3.12)*	− 0.57	− 6.00 (8.52)	− 4.50 (− 6.67, − 2.32)*	− 0.47
	2-months	− 5.50 (5.60)	0.00 (− 2.24, 2.24)	0.00	− 3.90 (7.36)	− 6.57 (− 8.75, − 4.38)*	− 0.78	− 5.25 (7.86)	− 5.25 (− 7.49, − 3.00)*	− 0.56
	Follow-up	− 4.75 (5.73)	− 0.75 (− 3.50, 2.00)	− 0.13	− 5.95 (10.5)	− 4.52 (− 7.21, − 1.83)*	− 0.45	− 5.25 (8.35)	− 5.25 (− 8.00, − 2.49)*	− 0.55
Wrist active extension	Baseline	19.25 (18.0)			17.57 (11.8)			13.10 (6.54)		
	1-month	19.85 (18.8)	− 0.60 (− 4.04, 2.84)	− 0.03	27.81 (15.2)	− 10.23 (− 13.59, − 6.88)*	− 0.77	17.25 (8.96)	− 4.15 (− 7.59, − 0.71)*	− 0.54
	2-months	20.25 (19.3)	− 1.00 (− 5.28, 3.28)	− 0.05	32.29 (17.1)	− 14.71 (− 18.9, − 10.5)*	− 1.03	21.50 (11.0)	− 8.40 (− 12.6, − 4.11)*	− 0.95
	Follow-up	22.00 (19.7)	− 2.75 (− 7.47, 1.97)	− 0.15	36.90 (17.3)	− 19.33 (− 23.9, − 14.72)*	− 1.54	21.00 (11.5)	− 7.90 (− 12.6, − 3.17)*	− 0.86
Wrist passive extension	Baseline	40.75 (16.6)			46.43 (12.5)			36.25 (14.7)		
	1-month	44.25 (13.8)	− 3.50 (− 6.95, − 0.04)*	− 0.23	57.38 (10.9)	− 10.95 (− 14.32, − 7.57)*	− 0.96	44.00 (13.6)	− 7.75 (− 11.20, − 4.29)*	− 0.56
	2-months	44.75 (13.7)	− 4.00 (− 7.40, − 0.59)*	− 0.27	60.24 (14.3)	− 13.81 (− 17.13, − 10.4)*	− 1.06	50.25 (11.9)	− 14.00 (− 17.40, − 10.5)*	− 1.07
	Follow-up	48.25 (14.6)	− 7.50 (− 10.42, − 4.5)*	− 0.49	60.95 (13.0)	− 14.52 (− 17.37, − 11.6)*	− 1.16	49.75 (13.6)	− 13.50 (− 16.42, − 10.6)*	− 0.98
MCP resting angle	Baseline	− 29.75 (17.2)			− 37.14 (13.9)			− 35.00 (15.6)		
	1-month	− 31.00 (16.3)	1.25 (− 2.14, 4.64)	0.07	− 30.24 (12.6)	− 6.90 (− 10.21, − 3.59)*	− 0.53	− 32.75 (13.1)	− 2.25 (− 5.64, 1.14)	− 0.16
	2-months	− 29.00 (15.4)	− 0.75 (− 3.58, 2.08)	− 0.04	− 29.05 (14.8)	− 8.09 (− 10.86, − 5.32)*	− 0.57	− 31.75 (13.7)	− 3.25 (− 6.08, − 0.41)*	− 0.22
	Follow-up	− 27.75 (15.4)	− 2.00 (− 6.12, 2.12)	− 0.12	− 29.05 (15.2)	− 8.09 (− 12.11, − 4.07)*	− 0.56	− 30.50 (14.0)	− 4.50 (− 8.62, − 0.38)*	− 0.31
MCP active extension	Baseline	12.75 (12.1)			15.24 (13.8)			6.00 (9.95)		
	1-month	12.75 (12.1)	0.00 (− 2.62, 2.62)	0.00	21.67 (11.5)	− 6.42 (− 8.99, − 3.86)*	− 0.52	9.25 (10.04)	− 3.25 (− 5.87, − 0.62)*	− 0.33
	2-months	13.75 (12.1)	− 1.00 (− 4.67, 2.67)	− 0.08	22.14 (11.1)	− 6.90 (− 10.49, − 3.31)*	− 0.56	10.50 (10.7)	− 4.50 (− 8.17, − 0.82)*	− 0.44
	Follow-up	15.50 (11.3)	− 2.75 (− 7.91, 2.41)	− 0.24	24.05 (10.0)	− 8.81 (− 13.84, − 3.76)*	− 0.75	11.25 (11.9)	− 5.25 (− 10.41, − 0.08)*	− 0.49

*SD* Standard deviation, *MD* Mean difference (baseline minus each time), *CI 95%* Confidence interval 95%, *ES* Effect size, *d* Cohen’s d.

*The difference is statistically significant p < .05.

### Effects on hand strength

A RM-MANOVA was calculated for the two measures of hand strength for the three groups in the four time points. The results of the multivariate effect of the interaction among these factors (hand strength, group and time) were statistically significant: Pillai’s trace = .252, F = 2.73, p = .016, η^2^ = .126. Once the RM-MANOVA gave significant results follow-up repeated measures ANOVAs were performed for each dependent variable. There were significant interaction (group x time) effects on hand grip strength (F = 2.55; p = .031; η^2^ = .08) as for the pinch strength (F = 3.29; p = .013; η^2^ = .10). There were gains in both experimental groups for the two dynamometric strength measurements, as it can be seen in the means and mean differences of Table 3. Additionally, post-hoc tests were performed via Bonferroni corrections. Regarding the 35 Hz group, there were statistically significant gains in both measures of strength among baseline and the three time moments (half treatment, end of treatment, and follow-up). In the case of 50 Hz group, there was no significant gain between 1 month and baseline in grip strength, but there were significant mean differences and large effects for the rest of comparisons. The largest difference was between baseline and follow-up for the 35 Hz NMES (Mean difference = 2.69 kg_f_, d = 0.56). Mean differences and Cohen’s d of all times against baseline are shown in Table 3. Looking at these effect sizes, the 50 Hz NMES had slightly largest effects than the 35 Hz NMES.

**Table 3:** Means, standard deviations, mean differences, confidence intervals and effect sizes of the variables related to the hand strength and the EMG activity in all the time points.

Measure		Control group (n = 20)			35 Hz NMES (n = 21)			50 Hz NMES (n = 20)		
Strength (kg_f_)	Testing time	Mean (SD)	MD (CI 95%)	ES (d)	Mean (SD)	MD (CI 95%)	ES (d)	Mean (SD)	MD (CI 95%)	ES (d)
Grip strength	Baseline	4.80 (6.30)			5.83 (4.70)			3.81 (3.01)		
	1-month	5.35 (7.05)	− 0.55 (− 1.66, 0.56)	− 0.08	7.00 (4.92)	− 1.16 (− 2.25, − 0.07)*	− 0.25	4.71 (3.31)	− 0.90 (− 2.01, 0.21)	− 0.29
	2-months	5.34 (6.31)	− 0.53 (− 1.67, 0.59)	− 0.08	7.71 (5.08)	− 1.88 (− 2.99, − 0.77)*	− 0.40	6.09 (3.37)	− 2.27 (− 3.41, − 1.13)*	− 0.73
	Follow-up	5.95 (7.15)	− 1.15 (− 2.38, 0.08)	− 0.17	8.52 (5.06)	− 2.69 (− 3.89, − 1.48)*	− 0.56	6.24 (3.68)	− 2.42 (− 3.66, − 1.18)*	− 0.74
Pinch strength	Baseline	2.09 (2.39)			2.44 (2.12)			1.67 (1.55)		
	1-month	2.31 (2.40)	− 0.22 (− 0.64, 0.19)	− 0.09	3.02 (2.04)	− 0.58 (− 0.99, − 0.17)*	− 0.28	2.36 (1.84)	− 0.69 (− 1.11, − 0.27)*	− 0.41
	2-months	2.69 (2.49)	− 0.60 (− 1.16, − 0.03)*	− 0.25	3.13 (1.97)	− 0.69 (− 1.24, − 0.13)*	− 0.34	2.71 (1.58)	− 1.05 (− 1.61, − 0.48)*	− 0.68
	Follow-up	2.39 (2.07)	− 0.30 (− 0.92, 0.32)	− 0.13	3.37 (2.01)	− 0.92 (− 1.53, − 0.32)*	− 0.46	2.96 (1.98)	− 1.29 (− 1.91, − 0.67)*	− 0.74

EMG activity	Testing time	Mean (SD)	MD (CI 95%)	ES (d)	Mean (SD)	MD (CI 95%)	ES (d)	Mean (SD)	MD (CI 95%)	ES (d)
Peak A extensors (µV)	Baseline	89.95 (112.86)			73.24 (42.40)			62.75 (52.77)		
	1-month	95.45 (105.88)	− 5.50 (− 16.60, 5.60)	− 0.05	102.62 (40.22)	− 29.38 (− 40.22, − 18.54)*	− 0.73	73.85 (55.49)	− 11.10 (− 22.20, 0.00)	− 0.21
	2-months	97.50 (104.31)	− 7.55 (− 22.41, 7.31)	− 0.07	116.52 (55.81)	− 43.28 (− 57.79, − 28.78)*	− 0.89	84.45 (51.09)	− 21.70 (− 36.56, − 6.83)*	− 0.43
	Follow-up	92.25 (103.84)	− 2.30 (− 20.60, 16.00)	− 0.02	130.48 (58.56)	− 57.23 (− 75.10, − 39.37)*	− 1.14	101.60 (87.80)	− 38.85 (− 57.15, − 20.54)*	− 0.55
ACR extensors (%)	Baseline	42.35 (12.05)			39.70 (12.65)			45.25 (12.52)		
	1-month	43.71 (8.67)	− 1.36 (− 7.54, 4.80)	− 0.13	33.89 (10.84)	5.80 (− 0.21, 11.82)	0.50	41.64 (9.49)	3.60 (− 2.56, 9.77)	0.33
	2-months	42.28 (9.54)	0.06 (− 5.52, 5.65)	0.00	30.86 (10.69)	8.83 (3.37, 14.29)*	0.77	35.07 (7.17)	10.18 (4.59, 15.77)*	1.02
	Follow-up	40.90 (10.58)	1.44 (− 4.27, 7.16)	0.13	31.12 (10.62)	8.58 (2.99, 14.16)*	0.75	34.90 (8.63)	10.34 (4.62, 16.06)*	0.98

*SD* Standard deviation, *MD* Mean difference (baseline minus each time), *CI 95%* Confidence interval 95%, *ES* Effect size, *d* Cohen’s d, *A* Amplitude, *ACR* Co− activation ratio of the antagonist; * The difference is statistically significant p < .05.

### Effects on muscle tone

A RM-MANOVA was calculated for the two indicators of muscle tone for the three groups in the four time points. The results of the multivariate effect of the interaction among these factors (muscle tone, group and time) were significant: Pillai’s trace = .474, F = 2.79, p = .002, η^2^ = .237. Afterwards, repeated measures ANOVAs were performed for each dependent variable. There were also some statistically significant group per time interactions on the Modified Ashworth Scale (MAS) for the wrist flexors (F = 7.35; p< .001; η^2^ = .20) and the flexors of the MCP of the fingers (F = 10.62; p< .001; η^2^ = .27). Gains were present in both treatment groups. Table 4 summarises these means and standard deviations. Post-hoc tests were performed. For the control group no pairs of time points were significantly different (p> .05), with the exception of a small improvement in wrist flexors between baseline and the follow-up. Regarding the experimental groups, there were statistically significant improvements among baseline and the 2-months and follow-up testing times for all dependent variables (wrist flexors and MCP flexors), and with large effect sizes. Mean differences and Cohen’s d of all times against baseline are shown in Table 4 for the three groups. A careful look at the effect sizes for 35 Hz vs. 50 Hz group shows that 35 Hz NMES had larger effects on muscle tone.

**Table 4:** Means, standard deviations, mean differences, confidence intervals and effect sizes of the variables related to muscle tone in all the time points.

Measure		Control group (n = 20)			35 Hz NMES (n = 21)			50 Hz NMES (n = 20)		
MAS	Testing time	Mean (SD)	MD (CI 95%)	ES (d)	Mean (SD)	MD (CI 95%)	ES (d)	Mean (SD)	MD (CI 95%)	ES (d)
Wrist flexors	Baseline	1.85 (0.75)			2.24 (0.77)			2.05 (0.99)		
	1-month	1.85 (0.75)	0.00 (− 0.23, 0.23)	0.00	1.71 (0.72)	0.52 (0.29, 0.75)*	0.73	1.85 (0.93)	0.20 (− 0.03, 0.43)	0.21
	2-months	1.75 (0.72)	0.10 (− 0.23, 0.43)	0.14	1.29 (0.85)	0.95 (0.62, 1,27)*	1.20	1.20 (0.70)	0.85 (0.51, 1.18)*	1.01
	Follow-up	1.45 (0.61)	0.40 (0.01, 0.78)*	0.60	1.14 (0.91)	1.09 (0.72, 1.46)*	1.34	1.05 (0.61)	1.00 (0.61, 1.38)*	1.25
MCP flexors	Baseline	1.35 (1.04)			1.81 (0.98)			1.65 (1.23)		
	1-month	1.30 (1.03)	0.05 (− 0.19, 0.29)	0.05	1.62 (1.12)	0.19 (− 0.04, 0.42)	0.18	1.15 (0.88)	0.50 (0.25, 0.74)*	0.48
	2-months	1.30 (1.03)	0.05 (− 0.23, 0.33)	0.05	1.00 (0.89)	0.81 (0.53, 1.08)*	0.88	0.95 (0.76)	0.70 (0.41, 0.98)*	0.70
	Follow-up	1.10 (0.85)	0.25 (− 0.04, 0.54)	0.27	0.90 (0.83)	0.90 (0.61, 1.19)*	1.10	0.95 (0.76)	0.70 (0.40, 0.99)*	0.70

*MAS* Modified Ashworth Scale, *MCP* Metacarpophalangeal, *SD* Standard deviation, *MD* Mean difference (baseline minus each time), *CI 95%* Confidence interval 95%, *ES* Effect size, *d* Cohen’s d; * The difference is statistically significant p < .05.

### Effects on muscle electrical activity

A RM-MANOVA was calculated for the two indicators of electromyographic (EMG) activity for the three groups in the four time points. The results of the multivariate effect of the interaction among these factors, peak amplitude extensors and the antagonist co-activation ratio (ACR) for the extensors, were statistically significant: Pillai’s trace = .603, F = 8.19, p< .001, η^2^ = .301. Repeated measures ANOVAs were performed for each dependent variable. Statistically significant changes were found for the values related to the peak EMG amplitude (F = 8.18; p< .001; η^2^ = .22) and the ACR assessing the performance of the wrist extensors (F = 4.12; p = .002; η^2^ = .12). Both experimental groups showed up improvements. Considering the post-hoc tests, the control group showed no significant gains in the three time points against baseline for the two EMG measures (p> .05). Regarding the experimental groups, there were statistically significant gains among baseline and the 2-months and follow-up testing times for the two dependent variables with large effect sizes. When we compared 35 Hz and 50 Hz effects, 35 Hz NMES had larger effects for peak EMG amplitude extensors while 50 Hz NMES had larger effects for ACR extensors. Mean differences and Cohen’s d of all times against baseline are shown in Table 3 for the three groups.

### Effects on functional outcome measures

A RM-MANOVA was calculated for the two functional measures for the three groups in the four time points. The results of the multivariate effect of the interaction among these factors, Barthel Index and Box and Block Test (BBT), were statistically significant: Pillai’s trace = .403, F = 4.79, p< .001, η^2^ = .201. Once the RM-MANOVA gave significant results follow-up repeated measures ANOVAs were performed for each dependent variable. There were no significant interaction (group x time) effects on BBT (F = 1.03; p = .38; η^2^ = .03). The Barthel Index showed a statistically significant interaction in favour of the NMES group of 35 Hz (F = 7.97; p< .001; η^2^ = .22). Mean differences and Cohen’s d of all times against baseline are shown in Table 5 for the three groups. The control and the 50 Hz groups did not significantly change compared to baseline. However, Barthel Index significantly improved with respect to baseline for the 35 Hz group and with large effects.

**Table 5:** Means, standard deviations, mean differences, confidence intervals and effect sizes of functional outcome measures in all the time points.

		Control group (n = 20)			35 Hz NMES (n = 21)			50 Hz NMES (n = 20)		
Measure	Testing time	Mean (SD)	MD (CI 95%)	ES (d)	Mean (SD)	MD (CI 95%)	ES (d)	Mean (SD)	MD (CI 95%)	ES (d)
BBT	Baseline	8.55 (10.05)			6.05 (7.71)			5.55 (5.52)		
	1-month	11.05 (13.20)	− 2.50 (− 4.97, − 0.02)*	− 0.28	9.57 (8.86)	− 3.52 (− 5.93, − 1.11)*	− 0.42	8.70 (9.11)	− 3.15 (− 5.62, − 0.67)*	− 0.29
	2-months	12.20 (12.95)	− 3.65 (− 7.11, − 0.18)*	− 0.31	11.62 (9.89)	− 5.57 (− 8.95, − 2.19)*	− 0.63	10.75 (9.51)	− 5.20 (− 8.66, − 1.73)*	− 0.66
	Follow-up	13.50 (14.04)	− 4.95 (− 9.85, − 0.04)*	− 0.40	13.90 (11.64)	− 7.85 (− 12.64, − 3.06)*	− 0.80	14.10 (13.63)	− 8.55 (− 13.45, − 3.64)*	− 0.82
Barthel Index	Baseline	58.25 (17.11)			60.71 (14.26)			59.50 (13.85)		
	1-month	60.75 (18.01)	− 2.50 (− 6.89, 1.89)	− 0.14	69.29 (9.78)	− 8.57 (− 12.86, − 4.28)*	− 0.70	62.25 (13.72)	− 2.75 (− 7.14, 1.64)	− 0.20
	2-months	63.00 (18.60)	− 4.75 (− 10.82, 1.32)	− 0.27	74.76 (13.08)	− 14.04 (− 19.97, − 8.12)*	− 1.02	63.50 (14.15)	− 4.00 (− 10.07, 2.07)	− 0.29
	Follow-up	64.50 (19.66)	− 6.25 (− 12.40, − 0.09)*	− 0.34	79.05 (13.10)	− 18.33 (− 24.33, − 12.32)*	− 1.34	64.25 (14.98)	− 4.75 (− 10.90, 1.40)	− 0.33

*BBT* Box and Block Test, *SD* Standard deviation, *MD* Mean difference (baseline minus each time), *CI 95%* Confidence interval 95%, *ES* Effect size; *d* Cohen’s d; * The difference is statistically significant p < .05.

### Analysis to determine the relationships between impairments and function

Post-stroke recovery is a complex process and can be viewed from different aspects (impairments, activity, and participation) according to the International Classification of Functioning, Disability and Health (ICF). Therefore, it could be of interest to test for potential patterns of relationships between impairments (range of motion, strength, tone, EMG parameters) and function (BBT and Barthel Index) from an ICF perspective. Accordingly, and as additional analyses, zero-order correlations were calculated among the 11 measures of motor impairment and the two functional outcome measures (BBT and Barthel Index), both in T1 (baseline) and T4 (follow-up). Correlations are shown in Table 6. There are a number of significant correlations among motor impairment and functional variables. In general, the correlations were much larger and consistent in Time 4.

**Table 6:** Correlations among the motor impairment variables and the functional outcome measures.

Variable	BBT		Barthel Index	
	Time 1	Time 4	Time 1	Time 4
Wrist resting angle	0.204	0.349**	0.083	0.266*
Wrist active extension	0.710**	0.492**	0.420**	0.652**
Wrist passive extension	0.197	0.190	0.086	0.436**
MCP resting angle	0.195	0.216	0.098	0.251
MCP active extension	0.318*	0.357**	0.369**	0.592**
Grip strength	0.541**	0.473**	0.172	0.429**
Pinch strength	0.557**	0.533**	0.313*	0.477**
MAS score for wrist flexors	− 0.219	− 0.321*	− 0.150	− 0.345**
MAS score for MCP flexors	− 0.366**	− 0.388**	− 0.187	− 0.395**
Peak EMG amplitude extensors	0.501**	0.414**	0.185	0.432**
ACR extensors	− 0.170	− 0.353**	− 0.116	− 0.516**

*p < .05; ** p < .01; *BBT* Box and Block Test, *MCP* Metacarpophalangeal; *MAS* Modified Ashworth Scale, *ACR* Co-activation ratio of the antagonist.

## Discussion

This trial may have two important contributions. On the one hand, it offers evidence of positive effects of NMES on motor recovery in older adults after stroke. On the other hand, NMES treatments with different frequency stimulation parameters were compared, which provided additional insights related to the optimisation of the applied protocol.

Regarding the effect of NMES intervention, variables related to hand motor impairment were studied, such as range of motion, hand strength, muscle tone, and muscle electrical activity. Both protocols applied produced improvements in wrist and finger joint movements. Evidence from this study is similar to that of the great majority of authors who have evaluated this outcome measure^30–35^. In contrast, there are also studies^36,37^, which were directed towards patients in the acute phase, that did not show significant findings. Considering the positive results demonstrated in various studies with very different NMES protocols, the stimulation parameters may not be crucial for range of motion gain, although the results of this research showed that the 35 Hz protocol had a larger effect on range of motion when it was compared with the 50 Hz NMES treatment.

Both studied protocols promoted improvements in the grip and pinch strength, although the 50 Hz NMES treatment had slightly larger effects than the 35 Hz NMES. These findings agree with the results of other authors^14,27,34,37,38^. Most of these studies mentioned selected patients with distal movement in the wrist or fingers. Referring to this point, Doucet and Griffin demonstrated differences according to the sample selected, where only patients with a high level of functionality showed changes^27^. In addition, these authors pointed out that a NMES group that used a stimulation frequency of 40 Hz showed significant improvements compared with a 20 Hz NMES group, concluding that higher frequencies could be more effective in improving strength. Previous studies also noted an increase in force production linked to the increase in NMES frequency. In this regard, Dreibati et al.^39^ showed that NMES of the quadriceps femoris muscle in healthy adults represented respectively 71, 62, 55% of maximum voluntary contraction force for stimulation frequencies of 100, 50 and 20 Hz. Increases in muscle strength through NMES programmes would require the maintenance of 60% maximum voluntary contraction^39^. On the other hand, studies such as those by Powell et al.^36^ and Chan et al.^33^ revealed no significant findings for hand strength when NMES was applied. Powell et al.^36^ pointed out that although a significant effect was not found, patients with a residual motor function showed better results. It should also be noted that the frequency used in this NMES protocol was 20 Hz, which could be a value with worse results in relation to strength, as concluded by Doucet and Griffin^27^. The study by Chan et al.^33^ addressed patients without residual distal movement and in the chronic phase. Moreover, the total dose of electrostimulation was very low in comparison with other studies (5 h), factors which could have a bearing on the lack of results. Having appraised the results of the different trials, it would seem that low stimulation frequencies around 20 Hz could be less efficient in improving strength, and also that sample characteristics related to the presence of residual distal movement could influence the effectiveness of the treatment.

Considering the MAS scale, both NMES protocols were shown to be effective in improving distal muscle tone in the upper limb, although the 35 Hz protocol had a larger effect when it was compared with the 50 Hz intervention. This effectiveness has also been reported by other authors^30,34,40^. However, there are also studies which failed to do so, such as those by Chan et al.^33^ and Powell et al.^36^, the features of which have already been mentioned.

Both NMES interventions were shown to improve muscle electrical activity in the wrist extensors, both in maximum activity and in the pattern of activation as antagonist. The few studies published in which EMG activity has been analysed^34^, used different EMG data collection protocols and although indications of improvement in muscle electrical activity were shown, comparison and the extraction of conclusive data on this point prove difficult. Doucet and Griffin studied the parameter of root mean square amplitude in the thumb adductor and obtained significant improvements in the group with high functionality and a greater effect with the NMES protocol which applied the stimulation frequency of 40 Hz compared to 20 Hz^27^. Some relationship was noted between change in co-activation and MAS scores, which could explain that improvements in muscle tone could also be associated to changes in neuromuscular control.

Post-stroke recovery is a complex process that needs to be addressed on different aspects (impairments, activity, and participation) according to the ICF. Different studies stated that recovery at the impairment and body structure levels constitutes the basis to further recovery of activity domain of the ICF classification^41–43^. Given the results obtained, we consider carrying out subsequent correlational analysis to elucidate if there was a pattern of relationships between impairments (range of motion, hand strength, tone, and EMG activity) and function (BBT and Barthel Index) from an ICF perspective.

Regarding functional measures, gains were evident only in the independence in activities of daily living (ADL), in favour of the 35 Hz NMES group. The results of this study showed that all three groups improved in manual dexterity, but although this improvement was greater in the experimental groups, these differences were not significant. The minimal clinically important difference (MICD) found in the literature of the Box and Block Test was 6 blocks/min for the more affected hand^44^. In our study the difference in manual dexterity did not show statistically significant differences between the groups because the three groups showed improvement. However, if we attend to the changes at MCID level, only the 35 Hz NMES group and the 50 Hz NMES group exceeded the MICD in the follow-up period (7.85 blocks and 8.55 blocks, respectively). The motor characteristics of the sample could have influenced these results. Alon et al. carried out two similar studies whose samples had different degrees of motor performance at the upper limb level^7,45^. Manual dexterity only improved in the study in which NMES intervention was applied in a sample with mild/moderate paresis^7^. This idea is also supported by Knutson et al. who indicated that the magnitudes of improvement were greater in participants with moderate hand impairment at baseline and a post-stroke period more than 6 months but less than 2 years of evolution^46^. Moreover, perhaps a longer duration of the treatment would have influenced the functional motor ability improvement, given the strong relationship between motor impairments and manual dexterity, as can be see in zero-order correlation analyses. In this regard, it has been hypothesized that 5 months would be necessary for an effective NMES intervention to improve upper limb function^47^. Although a NMES protocol with active participation of the subjects was proposed, more cognitive effort and goal-driven functional tasks could have also influenced the results, since they are elements that could be crucial in promoting neuroplasticity and achieving functional results^17^. Finally, the stimulation frequency could also have an impact on function. In relation to this, Doucet and Griffin only found improvement in manual dexterity in the high functioning group (Fugl-Meyer Motor Assessment score greater than 60) and with low frequency stimulation programmes around 20 Hz^27^. Subsequent analyses were carried out to determine which of the improved impairments best explained changes in Barthel Index. In general, the correlations were much larger and consistent in the follow-up period (T4) when they were compared with baseline (T1), which is reasonable given that most motor impairment measures improved with NMES and therefore there was more variability in the sample. The motor impairments that at baseline did not show significant correlations, but that at the end of the study presented associations with the Barthel Index were wrist resting angle, wrist passive extension for range of motion, grip strength, MAS, and EMG activity variables.

This study is the first randomised clinical trial (RCT) of this nature aimed at older adults. The NMES protocols proposed show evidence of improvements in this subpopulation, but it remains unknown whether individuals of another age range would have responded in the same way to the treatment. Aging produces a series of changes associated with the central nervous system and the musculoskeletal system^48^, among others, which could condition a specific response to NMES intervention. The RCTs reviewed present heterogeneous samples and there is a lack of studies investigating the older population, or analyzing results according to age. Doucet and Griffin showed that there are differences in muscle response according to the different stimulation patterns during fatigue NMES protocols that could be related to changes in the neuromuscular system caused by central paralysis and age^28^. In addition, they pointed out the need to define specific stimulation parameters that maximise force output and delay the onset of fatigue in the clinical applications of NMES in the post-stroke population^28^.

The optimum stimulation parameters are currently unknown^17^. Research indicated that frequency (number of pulses per second) affect force and fatigue development and that there is difference between muscles of different fibre composition (type I and type II fibres) and size, being necessary to adjust this parameter in each NMES programme^49^. In the present study, two NMES treatments with different stimulation frequency were compared. Both NMES protocols showed evidence of improvements in motor impairment variables, although the 35 Hz intervention had larger effects on range of motion, muscle tone and peak EMG amplitude of wrist extensors; and the 50 Hz NMES intervention had a larger effect on hand strength and the ACR of wrist extensors. With regard to the functional measures, only 35 Hz NMES intervention proved to be effective in functional independence in ADL. No significant gain between-groups was obtained related to manual dexterity. Only two articles have been found which study in depth this parameter; thus Doucet and Griffin showed that stimulation programmes that included higher frequencies around 40 Hz and varying pulse patterns were more effective in maximizing force output than lower frequencies around 20 Hz constant-pattern stimulation programmes^28^. The same authors later published another study in which the muscles of the thenar eminence were stimulated with the aim of investigating fine motor control, comparing protocols with frequencies of 20 Hz versus 40 Hz^27^. These authors concluded that the specific stimulation frequencies selected may have a direct impact on skills gained. Their results suggested that stimulation frequencies in the region of 40 Hz might be more effective for improving strength and motor activation properties, while lower levels around 20 Hz could have a greater impact on manual dexterity and muscular endurance^27^. In addition, the frequency could be related to particular patterns of muscle fibre activation. Frequencies below 40–50 Hz recruited more slow-twitch (type I fibres), while higher frequencies recruited more fast-twitch (type IIa and IIb fibres)^39^. Spasticity, lack of activity and disuse following stroke produce adaptive changes in the anatomy, biomechanics, and functionality of the nervous and musculoskeletal systems. Among these changes, Edstrom et al.^50^ and Dattola et al.^51^ made reference to modifications in the type of muscle fibre, characterized by a greater predominance of type I fibres and reduction in the proportion of type II fibres, a change also contributed to by aging^29,48^. These type I muscle fibres could have a greater affinity for stimulation frequencies below 40–50 Hz^39^. A current study concluded that if the main function of the muscle is related to sustained repetition of fine motor movements, lower-moderate NMES frequencies are preferred. By contrast, muscle functions related to force generation would require higher frequencies to achieve force production near of 60% maximum voluntary contraction^49^. Further research is needed to study this subject in depth to provide more solid data.

Despite the fact that the design of this study is an RCT, certain limitations must be borne in mind such as the lack of blinding of the physical therapists who applied the treatment and of the assessor, the relatively short follow-up period, the retrospective registration of the RCT, and the difficulties in extrapolating results to other age groups. In addition, peak amplitude used as an EMG parameter is a measure than could be biased by the noise level. Advances in neuroscience and new technologies may possibly give rise to new applications of NMES for upper limb recovery after stroke, but future NMES protocols should take into account whether the basic programming parameters of the equipment are clinically relevant. More research is needed to elucidate the specific mechanism of action associated with the different stimulation parameters in order to guide clinical decisions. Elements such as motor characteristics of the subject, treatment dose, stage after stroke, cognitive effort, goal-driven functional tasks, and a proper selection of stimulation frequency according to the specific deficits should be addressed in the design of a NMES protocol. Future studies should analyse these aspects in depth in order to adapt treatment resources to the needs of each patient, and subsequently to optimise individual interventions and to achieve larger treatment effects.

In conclusion, the NMES intervention could be a useful complementary treatment for upper limb motor recovery in older adults with spastic hemiparesis following stroke. Both NMES protocols showed evidence of improvements in measurements related to hand motor impairment, but nevertheless, effect sizes revealed different importance in range of motion, hand strength, muscle tone, and muscle electrical activity of the wrist extensors. Regarding functional improvements, only a superior effect for the 35 Hz NMES protocol in functional independence was found. These findings showed that the stimulation frequency selected could have different effects depending on skill under study.

## Methods

### Study design and participants

This study was a single-blind RCT where the participants were blind to group assignment. Participants were randomly assigned to the three groups: two experimental groups (50 Hz NMES and 35 Hz NMES) and a control group. A statistician not involved with the intervention or data collection performed the random allocation sequence (random number generator of SPSS 22). There were no restrictions in the randomisation. A priori power analyses were performed and the sample size needed for a power of .85, with the usual alpha of .05, and an effect size of d =1 as estimated in the meta-analysis by Pomeroy et al.^24^ was 20 subjects per group.

The study was approved by the Scientific and the Ethics Committee for Clinical Research of the Doctor Peset University Hospital of Valencia, Spain (CC 43/09) and was registered in the ClinicalTrials.gov (identifier NCT03913624; 12/04/2019). This study was retrospectively registered given that when the experiment was being performed pre-registration was not mandatory. The procedures were conducted according to the Declaration of Helsinki. All participants in the research were informed and signed informed consent prior to study participation. The Consolidated Standards of Reporting Trials (CONSORT) checklist was used to report the RCT.

Participants were recruited from the aforementioned hospital, who attended for physical therapy intervention as outpatients between July 2009 and September 2014. A total of 262 older adults affected by spastic hemiparesis of the hand after stroke were screened for study eligibility. Of these, 69 individuals were scheduled for a baseline assessment. A flow diagram describes the participant eligibility and randomisation of the selected participants (see Fig. 1). The inclusion criteria were presence of spastic hemiparesis caused by stroke (diagnosed by neuroimaging tests), a score ≤ 3 on the MAS for wrist and finger flexors, residual voluntary movement of wrist (active wrist extension ≥ 5° from the resting position) , wrist extension response to stimulation, age ≥ 60 years, post-stroke period < 18 months, clinical stability, and MMSE score ≥ 23 with the absence of significant cognitive impairment, being able to follow basic instructions and to collaborate in the treatment. The spasticity assessment included the Tardieu Scale and hyperreflexia of the deep tendon reflexes. Exclusion criteria comprised those situations that could alter the results or posed a risk for the patient. Table 7 displays the exclusion criteria taken into account in the study.

**Table 7:** Exclusion criteria.

Dermatological reactions with the application of stimulation
Significant sensory deficits in the affected arm
Previous musculoskeletal problems of the hand
Treatment with the botulin toxin
Anti-spastic medication usage
Cardiac pacemaker, implanted electronic device, or metal implants in the affected arm
Complex regional pain syndrome
Severe aphasia, history of epileptic seizures, psychiatric disorder, or important alterations of behavior
Severe visual impairment
Any comorbid neurological disease
Important deformity or obesity that affects the application of the NMES
Potentially fatal cardiac arrhythmia or other descompensated heart disease
Systemic infectious process, cancer, or other terminal disease

### Outcome measurements

Shoulder motor control was measured with a reduced version of the MESUPES-arm test subscale within the Motor Evaluation Scale for Upper Extremity in Stroke patients^52^.

A universal goniometer was used to measure the resting angle, active extension and passive extension of the wrist (°), and a special JAMAR finger goniometer to evaluate the resting angle and active extension of the MCP of the fingers (°).

The grip strength was assessed by means of a standardised hydraulic hand dynamometer (JAMAR brand, model 5030J1, Sammons and Preston INC) and the pinch strength using a standardised hydraulic pinch gauge (JAMAR brand, model 7498-05, Sammons and Preston INC). Unit of measurement was kg_f_. Three repetitions were done in both evaluations, taking the highest value and leaving a resting period of 1 min between them.

Muscle tone of the wrist flexors and the MCP flexors of the fingers was measured using MAS^53,54^. The assessment was started 5 min after laying the subject down in the supine position^54^ and using a single passive movement to evaluate each muscle group^55^. Joints were moved from maximum possible flexion to maximum possible extension over a duration of about 1 s by counting one thousand one^53,54,56^ to standardise the speed of the movement being tested.

EMG activity was recorded in the radial extensor and the radial flexor of the carpus using the Muscle Trainer (model METR-0, Mega Electronics Ltd, Kuopio, Finland) and circular adhesive surface bipolar electrodes of Ag/AgCl (Ambú Blue Sensor, diameter 10mm). EMG preamplifier measurement sensitivity is ± 1 µV. The measurement range for RMS EMG signals was 0–4095 µV. AD-transformer for each EMG channel was carried out with an accuracy of 12 bits. Data memory was 32 kB. Signals were band-pass filtered at 20–500 Hz. Data was transferred from the receiver to a personal computer and analysed using MegaWin v.2.0 (Mega Electronics Ltd, Kuopio, Finland). The patient was asked to perform maximum voluntary isometric contractions (MVICs) of both the flexors and extensors of the wrist for 5 s against manual resistance, and the electrical activity of both the agonist and antagonist muscles was recorded simultaneously. EMG parameters were calculated related to the peak amplitude (in µV) and the antagonist co-activation ratio (ACR) calculated during MVICs of the wrist flexors (ACR = antagonist activity/ [agonist activity + antagonist activity] × 100%)^57^. ACR provides an estimate of the relative activation of the agonist-antagonist muscle pair and the magnitude of the co-activation of the antagonist. The average amplitude of the extensors was considered as the antagonist activity (µV) and the average amplitude of the flexors was considered as the agonist activity (µV).

Manual dexterity was assessed with BBT^58^ scored by counting the number of blocks moved by the affected side from one compartment of a box to another within 1-min trial period, and functional independence in ADL by Barthel Index^59^.

There were four measurements over time: pre-treatment (T1), one month from the beginning of the study (T2), two months from the beginning of the study (end of treatment) (T3), and three months from the beginning of the study (follow-up) (T4). The second and third assessments were performed within 48 and 72 h after the last application of electrostimulation and were always recorded in the morning, in the same order and by the same examiner.

### Interventions

During an 8-week intervention period, training was conducted for 3 days per week (a total of 24 sessions). The two experimental groups received the conventional treatment (the same as the control group) for the same amount of time, plus NMES. The NMES application time was 20 min for the first 2 sessions and 30 min for subsequent sessions. Each NMES session took place under the supervision of an experienced physical therapist.

50 Hz NMES group: NMES was applied on wrist and finger extensors. The main electrostimulation parameters consisted of low-frequency current, a stimulation frequency of 50 Hz, symmetrical rectangular biphasic wave, and pulse duration of 300 μs.

35 Hz NMES group: NMES was applied on wrist and finger extensors. The main electrostimulation parameters consisted of low-frequency current, a stimulation frequency of 35 Hz, symmetrical rectangular biphasic wave, and pulse duration of 300 μs.

The electrostimulation programmes were only differentiated in the parameter of the stimulation frequency, 35 Hz or 50 Hz, depending on the experimental group to which the patient belonged. The rest of the parameters were the same. The intensity was adjusted in order to allow a maximum extension of wrist and fingers ensuring the patient's comfort. Ramping up/down periods were established at a time of 2 s during the first week, and 1 s for the rest of the study. The contraction-relaxation times were adjusted during the treatment period (5–25 s in the first 2 weeks, 5–20 s in the third week, 5–15 s in the fourth week, 5–10 s during fifth to sixth weeks, and 5–5 s in final weeks). These parameters were modified during the treatment in order to adapt the training progressively and avoid muscle fatigue^60,61^. The application time was 20 min for the first two sessions and 30 min for subsequent sessions. Three sessions per week were conducted for a period of 8 weeks. Additionally, the patient was asked to actively participate by means of a voluntary contraction on feeling the stimulus and visualizing the movement.

The electrodes were placed over the extensor muscles of the wrist and fingers, stimulating mainly the extensor carpi radialis longus and brevis, and the extensor digitorum communis. A line of the humeral epicondyle was drawn on the posterior part of the forearm to the midpoint of the wrist joint, and this was divided into three parts, placing one electrode approximately in the proximal third of this described line, and the other electrode in the distal third towards the posterolateral side of the forearm. A good extensor response was sought and the individual placement was recorded by means of a metric tape for later reproduction. The participant was placed in a sitting position (shoulder abduction of 0–30°, elbow flexion of 70–90°, pronated forearm, with a towel at the distal level of the forearm to start with a slight wrist flexion)^62^.

For the application of the NMES, a portable apparatus (Beac Medical IntelliSTIM^®^ BE 28-E) and disposable self-adhesive surface electrodes (En-Trode^®^ 50 × 50 mm) were used. The application of the NMES was tested to rule out any abnormal reaction to the passage of current or to the material used. The state of the skin and the presence of pain were assessed. The output frequency of the equipment was verified through oscilloscope tests.

The control group received standard physical therapy intervention in the reference rehabilitation centre. Two physical therapists with extensive expertise applied the conventional treatment. Each session lasted approximately 60 min with the following structure: (1) Warm-up with cycle ergometer,10 min; (2) Stretching (20 s/2–3 repetitions) and passive/active-assisted upper and lower limb kinesiotherapy (3 series/10–15 repetitions), 10 min; (3) Bimanual exercises (e.g., task-specific exercises such as gripping and releasing objects, shoulder pulley, and elastic band training), 10 min; (4) Mobility and strengthening lower limb exercises (2–3 series, 10–15 repetitions), 10 min; (5) Coordination, balance and gait training, 20 min. The exercises were progressively adapted depending on the degree of motor function of the patient.

### Statistical analyses

Statistical analyses included descriptive statistics and inferential analyses for group comparisons. Groups under study were compared in the four time points in several outcomes. Firstly, the groups under study were compared in their basal (pre-treatment) scores using one-way ANOVAs for quantitative variables and chi-square tests of independence for the qualitative ones. Secondly, several RM-MANOVAs were performed for each of the blocks of dependent variables: range of motion, hand strength, muscle tone, EMG activity, and motor function. Thirdly, when the RM-MANOVA resulted in a significant interaction of group and time in the dependent variables, follow-up mixed 3 (group)  ×  4 (time) ANOVAs were employed. In order for the treatments to be considered effective, the group × time interaction on the dependent variables should be statistically significant (p< .05) with control versus treatment differences. Compliance with parametric assumptions was ensured before ANOVAs were estimated. Independence of observations was assured by design (randomization). The assumptions of normality of residuals, constancy of the variance and sphericity were tested by plotting standardised residuals against factor levels, residuals vs. fitted values, and Q–Q plots of residuals, and testing the Sphericity assumption with Mauchly’s test. These analyses found that lack of normality or unequal group variances did not jeopardized the parametric analyses, and wherever the Sphericity assumption was not met the correction by Huyhn and Felt was employed. Additionally to statistical significance, effect sizes were calculated for all test statistics, specifically partial eta-squares were estimated (η^2^) for the effects and Cohen’s d for the comparisons of all pairs of means. Significant interactions were further scrutinised with mean comparisons with Bonferroni corrections. Thirdly, zero-order correlations were calculated among the 11 measures of motor impairment and the two functional measures (BBT and Barthel Index).

## Data Availability

Study data are available from the corresponding author on reasonable request. The datasets generated during the current study are not publicly available due to legal and ethical restraints. Sharing of individual participant data was not included in the informed consent of the study, and there is potential risk of revealing participants’ identities as it is not possible to completely anonymize the data.

## References

[1] Johnson W, Onuma O, Owolabi M, Sachdev S (2016). Stroke: A global response is needed. Bull World Health Organ..

[2] Masjuan J (2011). Stroke health care plan (ICTUS II. 2010). Neurol. Engl. Ed..

[3] Benjamin EJ (2017). Heart disease and stroke statistics-2017 update: A report from the American Heart Association. Circulation.

[4] Kimberley TJ (2004). Electrical stimulation driving functional improvements and cortical changes in subjects with stroke. Exp. Brain Res..

[5] Narai E, Hagino H, Komatsu T, Togo F (2016). Accelerometer-based monitoring of upper limb movement in older adults with acute and subacute stroke. J. Geriatr. Phys. Ther..

[6] Kowalczewski J, Gritsenko V, Ashworth N, Ellaway P, Prochazka A (2007). Upper-extremity functional electric stimulation-assisted exercises on a workstation in the subacute phase of stroke recovery. Arch. Phys. Med. Rehabil..

[7] Alon G, Levitt AF, McCarthy PA (2007). Functional electrical stimulation enhancement of upper extremity functional recovery during stroke rehabilitation: A pilot study. Neurorehabil. Neural Repair..

[8] Hlustík Petr P, Mayer Michal M (2006). Paretic hand in stroke: From motor cortical plasticity research to rehabilitation. Cogn. Behav. Neurol..

[9] Howlett OA, Lannin NA, Ada L, McKinstry C (2015). Functional electrical stimulation improves activity after stroke: A systematic review with meta-analysis. Arch. Phys. Med. Rehabil..

[10] Monte-Silva K (2019). Electromyogram-related neuromuscular electrical stimulation for restoring wrist and hand movement in poststroke hemiplegia: A systematic review and meta-analysis. Neurorehabil. Neural Repair..

[11] Stein C, Fritsch CG, Robinson C, Sbruzzi G, Plentz RD (2015). Effects of electrical stimulation in spastic muscles after stroke: Systematic review and meta-analysis of randomized controlled trials. Stroke.

[12] Yang JD (2019). Effectiveness of electrical stimulation therapy in improving arm function after stroke: A systematic review and a meta-analysis of randomised controlled trials. Clin. Rehabil..

[13] Dobkin BH, Dorsch A (2013). New evidence for therapies in stroke rehabilitation. Curr. Atheroscler. Rep..

[14] De Kroon JR, Ijzerman MJ, Lankhorst GJ, Zilvold G (2004). Electrical stimulation of the upper limb in stroke: Stimulation of the extensors of the hand vs. alternate stimulation of flexors and extensors. Am. J. Phys. Med. Rehabil..

[15] Ring H, Rosenthal N (2005). Controlled study of neuroprosthetic functional electrical stimulation in sub-acute post-stroke rehabilitation. J. Rehabil. Med..

[16] Glanz M, Klawansky S, Stason W, Berkey C, Chalmers TC (1996). Functional electrostimulation in poststroke rehabilitation: A meta-analysis of the randomized controlled trials. Arch. Phys. Med. Rehabil..

[17] Quandt F, Hummel FC (2014). The influence of functional electrical stimulation on hand motor recovery in stroke patients: A review. Exp. Transl. Stroke Med..

[18] Chipchase LS, Schabrun SM, Hodges PW (2011). Peripheral electrical stimulation to induce cortical plasticity: A systematic review of stimulus parameters. Clin. Neurophysiol..

[19] Chae J, Sheffler L, Knutson J (2008). Neuromuscular electrical stimulation for motor restoration in hemiplegia. Top. Stroke Rehabil..

[20] Eraifej J (2017). Effectiveness of upper limb functional electrical stimulation after stroke for the improvement of activities of daily living and motor function: A systematic review and meta-analysis. Syst. Rev..

[21] Nascimento LR (2014). Cyclical electrical stimulation increases strength and improves activity after stroke: A systematic review. J. Physiother..

[22] Farmer SE, Durairaj V, Swain I, Pandyan AD (2014). Assistive technologies: Can they contribute to rehabilitation of the upper limb after stroke?. Arch. Phys. Med. Rehabil..

[23] Sentandreu-Mañó T, Salom Terrádez JR, Tomás JM, Company José C (2016). Evidence relating to the use of distal neuromuscular electrical stimulation in the recovery of stroke patients: A systematic review. Fisioterapia..

[24] Pomeroy VM, King L, Pollock A, Baily-Hallam A, Langhorne P (2006). Electrostimulation for promoting recovery of movement or functional ability after stroke. Cochrane Database Syst. Rev..

[25] Sluka KA, Walsh D (2003). TENS: Basic science mechanisms and clinical effectiveness. J. Pain..

[26] Diéguez-Pérez I, Leirós-Rodríguez R (2020). Effectiveness of different application parameters of neuromuscular electrical stimulation for the treatment of dysphagia after a stroke: A systematic review. J. Clin. Med..

[27] Doucet BM, Griffin L (2013). High-versus low-frequency stimulation effects on fine motor control in chronic hemiplegia: A pilot study. Top Stroke Rehabil..

[28] Doucet BM, Griffin L (2009). Variable stimulation patterns for poststroke hemiplegia. Muscle Nerve..

[29] Salech F, Jara R, Michea L (2012). Physiological changes associated with normal aging. Rev. Med. Clin. Condes..

[30] Sahin N, Ugurlu H, Albayrak I (2012). The efficacy of electrical stimulation in reducing the post-stroke spasticity: A randomized controlled study. Disabil Rehabil..

[31] Malhotra S (2013). A randomized controlled trial of surface neuromuscular electrical stimulation applied early after acute stroke: Effects on wrist pain, spasticity and contractures. Clin. Rehabil..

[32] Gabr U, Levine P, Page SJ (2005). Home-based electromyography-triggered stimulation in chronic stroke. Clin. Rehabil..

[33] Chan MK, Tong RK, Chung KY (2009). Bilateral upper limb training with functional electric stimulation in patients with chronic stroke. Neurorehabil. Neural Repair..

[34] Boyaci A (2013). Comparison of the effectiveness of active and passive neuromuscular electrical stimulation of hemiplegic upper extremities: A randomized, controlled trial. Int. J. Rehabil. Res..

[35] Gharib NM, Aboumousa AM, Elowishy AA, Rezk-Allah SS, Yousef FS (2015). Efficacy of electrical stimulation as an adjunct to repetitive task practice therapy on skilled hand performance in hemiparetic stroke patients: A randomized controlled trial. Clin. Rehabil..

[36] Powell J, Pandyan AD, Granat M, Cameron M, Stott DJ (1999). Electrical stimulation of wrist extensors in poststroke hemiplegia. Stroke.

[37] Rosewilliam S, Malhotra S, Roffe C, Jones P, Pandyan AD (2012). Can surface neuromuscular electrical stimulation of the wrist and hand combined with routine therapy facilitate recovery of arm function in patients with stroke?. Arch. Phys. Med. Rehabil..

[38] De Kroon JR, Ijzerman MJ (2008). Electrical stimulation of the upper extremity in stroke: cyclic versus EMG-triggered stimulation. Clin. Rehabil..

[39] Dreibati B, Lavet C, Pinti A, Poumarat G (2010). Influence of electrical stimulation frequency on skeletal muscle force and fatigue. Ann. Phys. Rehabilit. Med..

[40] Popovic MB, Popovic DB, Sinkjaer T, Stefanovic A, Schwirtlich L (2003). Clinical evaluation of functional electrical therapy in acute hemiplegic subjects. J. Rehabil. Res. Dev..

[41] Patel P, Kaingade SR, Wilcox A, Lodha N (2020). Force control predicts fine motor dexterity in high-functioning stroke survivors. Neurosci. Lett..

[42] Lin IH (2019). Effectiveness and superiority of rehabilitative treatments in enhancing motor recovery within 6 months poststroke: A systemic review. Arch. Phys. Med. Rehabil..

[43] Harris JE, Eng JJ (2007). Paretic upper-limb strength best explains arm activity in people with stroke. Phys. Ther..

[44] Chen HM, Chen CC, Hsueh IP, Huang SL, Hsieh CL (2009). Test-retest reproducibility and smallest real difference of 5 hand function tests in patients with stroke. Neurorehabil. Neural Repair..

[45] Alon G, Levitt AF, McCarthy PA (2008). Functional electrical stimulation may modify the poor prognosis of stroke survivors with severe motor loss of the upper extremity: A preliminary study. Am. J. Phys. Med. Rehabil..

[46] Knutson JS, Gunzler DD, Wilson RD, Chae J (2016). Contralaterally controlled functional electrical stimulation improves hand dexterity in chronic hemiparesis: A randomized trial. Stroke.

[47] Lourenção MI, Battistella LR, Martins LC, Litvoc J (2005). Analysis of the results of functional electrical stimulation on hemiplegic patients’ upper extremities using the Minnesota manual dexterity test. Int. J. Rehabil. Res..

[48] Sions JM, Tyrell CM, Knarr BA, Jancosko A, Binder-Macleod SA (2012). Age- and stroke-related skeletal muscle changes: A review for the geriatric clinician. J. Geriatr. Phys. Ther..

[49] Vromans M, Faghri PD (2018). Functional electrical stimulation-induced muscular fatigue: Effect of fiber composition and stimulation frequency on rate of fatigue development. J. Electromyogr. Kinesiol..

[50] Edstrom L, Grimby L (1986). Effect of exercise on the motor unit. Muscle Nerve..

[51] Dattola R (1993). Muscle rearrangement in patients with hemiparesis after stroke: An electrophysiological and morphological study. Eur. Neurol..

[52] Van de Winckel A (2006). Can quality of movement be measured? Rasch analysis and inter-rater reliability of the motor evaluation scale for upper extremity in stroke patients (MESUPES). Clin. Rehabil..

[53] Bohannon RW, Smith MB (1987). Inter-rater reliability of a modified Ashworth scale of muscle spasticity. Phys Ther..

[54] Gregson JM (1999). Reliability of the tone assessment scale and the modified Ashworth scale as clinical tools for assessing poststroke spasticity. Arch. Phys. Med. Rehabil..

[55] Nuyens G (1994). Interrater reliability of the Ashworth Scale in multiple sclerosis. Clin. Rehabil..

[56] Mehrholz J (2005). Reliability of the modified tardieu scale and the modified ashworth scale in adult patients with severe brain injury: A comparison study. Clin. Rehabil..

[57] Silva CC (2014). Co-activation of upper limb muscles during reaching in post-stroke subjects: An analysis of the contralesional and ipsilesional limbs. J. Electromyogr. Kinesiol..

[58] Mathiowetz V, Volland G, Kashman N, Weber K (1985). Adult norms for the box and block test of manual dexterity. Am. J. Occup. Ther..

[59] Baztán JJ (1993). The Barthel index: A validated instrument for the functional assessment of stroke patients. Rev. Esp. Geriatr. Gerontol..

[60] Packman-Braun R (1988). Relationship between functional electrical stimulation duty cycle and fatigue in wrist extensor muscles of patients with hemiparesis. Phys. Ther..

[61] Cauraugh JH, Kim SB (2003). Chronic stroke motor recovery: Duration of active neuromuscular stimulation. J. Neurol. Sci..

[62] Cameron T, McDonald K, Anderson L, Prochazka A (1999). The effect of wrist angle on electrically evoked hand opening in patients with spastic hemiplegia. IEEE Trans. Rehabil. Eng..

